# Is Glioblastoma an Epigenetic Malignancy?

**DOI:** 10.3390/cancers5031120

**Published:** 2013-09-03

**Authors:** Marta Maleszewska, Bozena Kaminska

**Affiliations:** Laboratory of Molecular Neurobiology, Neurobiology Center, The Nencki Institute of Experimental Biology, 3 Pasteur Str., Warsaw 02-093, Poland

**Keywords:** epigenetics, methylator phenotype, histone modifying enzymes, inhibitors and glioblastoma

## Abstract

Epigenetic modifications control gene expression by regulating the access of nuclear proteins to their target DNA and have been implicated in both normal cell differentiation and oncogenic transformation. Epigenetic abnormalities can occur both as a cause and as a consequence of cancer. Oncogenic transformation can deeply alter the epigenetic information enclosed in the pattern of DNA methylation or histone modifications. In addition, in some cancers epigenetic dysfunctions can drive oncogenic transformation. Growing evidence emphasizes the interplay between metabolic disturbances, epigenomic changes and cancer, *i.e.*, mutations in the metabolic enzymes SDH, FH, and IDH may contribute to cancer development. Epigenetic-based mechanisms are reversible and the possibility of “resetting” the abnormal cancer epigenome by applying pharmacological or genetic strategies is an attractive, novel approach. Gliomas are incurable with all current therapeutic approaches and new strategies are urgently needed. Increasing evidence suggests the role of epigenetic events in development and/or progression of gliomas. In this review, we summarize current data on the occurrence and significance of mutations in the epigenetic and metabolic enzymes in pathobiology of gliomas. We discuss emerging therapies targeting specific epigenetic modifications or chromatin modifying enzymes either alone or in combination with other treatment regimens.

## 1. Nature of Epigenetic Modifications

The extent of chromatin condensation at each locus plays an important role in regulating the access of nuclear proteins to their target sites in DNA and hence directly influences cell functions [1]. The state of chromatin at a particular gene was found to be crucial for cell-fate decision making processes and has been implicated in both normal cell differentiation and oncogenic transformation. Remodeling of chromatin can be achieved in several, interconnected ways, including covalent modifications of histones and DNA methylation. Covalent modifications in DNA and chromatin structure constitute an “epigenetic code” [2] which is superimposed upon the genetic information contained within DNA and serves to regulate gene activity both during development and in a number of pathological situations, including cancer.

### 1.1. Role of Histone Modifications in Gene Regulation

**Figure 1:**
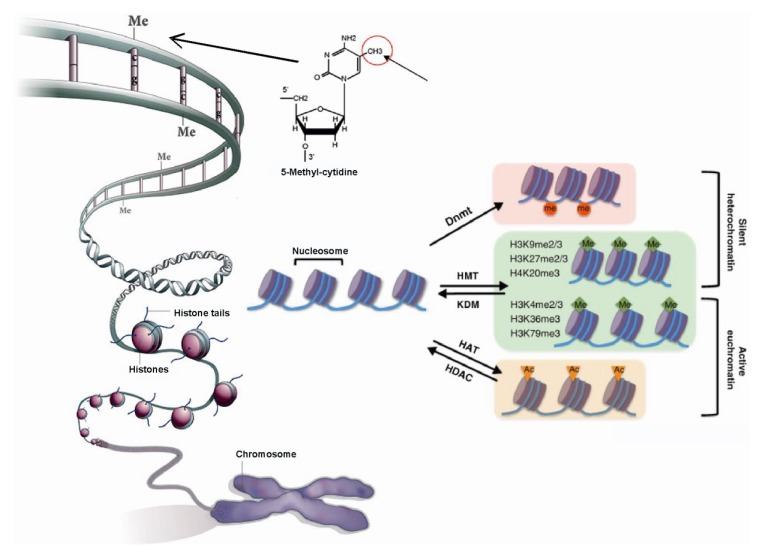
Role of epigenetic modification in chromatin organization.

Numerous covalent histone modifications at active or inactive loci, such as acetylation, methylation, phosphorylation and ubiquitination, notably at the *N*-terminal tails, have been described. For example, acetylation of *N*-terminal lysine residues of histones H3 and H4 is associated with active chromatin, while methylation of lysines 9 and 27 of histone H3 appears to be the hallmark of condensed chromatin at silent loci [2,3,4] (Figure 1). Histone modifications exert their effects via two main mechanisms. The first involves the modification(s) directly influencing the overall structure of chromatin. Histone acetylation and phosphorylation effectively reduce the positive charge of histones, and this has the potential to disrupt electrostatic interactions between histones and DNA. Notably, acetylation occurs on numerous histone tail lysines, including H3K9, H3K14, H3K18, H4K5, H4K8 and H4K12 [5]. Also single-site modifications (*i.e.*, H4K16ac) could be associated with gross structural chromatin changes [6]. Histone phosphorylation tends to be very site-specific and there are far fewer sites compared with acetylated sites. For instance, phosphorylation of H3S10 occurs genome-wide during mitosis and is associated with chromatin condensation [7]. Unlike acetylation and phosphorylation, methylation does not alter the overall charge of the molecule [8] but regulates (either positively or negatively) binding of effector molecules. Current evidence indicates that these modifications recruit transcription factors, chromatin remodelers or chromatin structure proteins involved in chromatin condensation or decondensation and contribute to the formation and maintenance of active or repressive chromatin states [9]. Numerous chromatin-associated factors can specifically interact with modified histones via many distinct domains such as bromodomain (*i.e.*, pCAF, BET proteins), chromodomain (*i.e.*, HP1, Suv39h1 and PcG proteins), MBT domain (*i.e.*, SFMBT), Tudor domain (*i.e.*, JMJD2A, SETDB1) or PhD finger (*i.e.*, HAT3, JARID1C) and others. However, histone modifications do not only function by providing dynamic binding platforms for various factors.

Histone modifications are regulated by action of pairs of opposing enzymes (Figure 1). Acetylation of lysines is regulated by histone acetyltransferases (HATs) and deacetylases (HDACs). HDAC enzymes oppose the effects of HATs and reverse lysine acetylation that restores the positive charge of the lysine and stabilizes the local chromatin architecture. HDACs are predominantly transcriptional repressors. There are 18 such enzymes identified, and these are subdivided into four major classes, depending on sequence homology. HDACs class I (HDAC 1–3 and HDAC8), class II (HDAC 4–7 and HDAC 9–10) and class IV (HDAC 11) share a related catalytic mechanism that requires a zinc ion. In contrast, class III HDACs (sirtuin 1–7) employ a distinct catalytic mechanism that is NAD^+^-dependent [10]. In general, HDACs have relatively low substrate specificity and the enzymes are typically present in multiple distinct complexes, often with other HDAC family members.

The best-characterized sites of histone methylation are those that occur on lysine residues. Lysines may be mono-, di- or tri-methylated. Although many lysine residues on the various histones are methylated, the best studied are H3K4, H3K9, H3K27, H3K36, H3K79, and H4K20 [5]. The first histone lysine methyltransferase (HKMT) to be identified was SUV39H1 that targets H3K9 [11]. Numerous HKMTs have since been identified, the vast majority of which methylate lysines within the *N*-terminal tails. HKMTs tend to be relatively specific enzymes (for example SUV39H targets H3K9 and MLL targets H3K4). Furthermore, HKMT enzymes also modify the appropriate lysine to a specific degree (*i.e.*, mono-, di- and/or tri-methyl state) (*i.e.*, SUV39H only di- and tri-methylates H3K9, and other enzyme G9a is responsible for H3K9 monomethylation) [12]. Nevertheless, all of the HKMTs that methylate *N*-terminal lysines contain a conserved SET domain that harbors the enzymatic activity. The only exception to this is hDOT1L, the enzyme that methylates H3K79 within the histone globular core. In any case, all HKMTs catalyse the transfer of a methyl group from *S*-adenosylmethionine (SAM) to a lysine’s ε-amino group [13].

The first lysine demethylase LSD1 was identified in 2004 [14]. LSD1 catalyses the removal of methyl groups from H3K4me1/2 using FAD as co-factor. Two years later another lysine demethylase JMJD2 that demethylates H3K9me3 and H3K36me3 was discovered [15]. The enzymatic activity of JMJD2 resides within a JmjC jumonji domain. Many histone lysine demethylases are now known and, except for LSD1, they all possess a catalytic Jumonji domain [12]. As with the lysine methyltransferases, the demethylases exhibit high substrate specificity with respect to their target lysine. They are also sensitive to the degree of lysine methylation; for instance, some of the enzymes are only capable of demethylating mono- and di-methyl substrates, whereas others can demethylate all three states of the methylated lysine.

### 1.2. Role of DNA Methylation in Gene Regulation

Methylation of DNA at cytosine residues within CpG dinucleotides occurs in genomes of most higher eukaryotes and is strongly correlated with transcriptional repression of imprinted genes as well as silencing of certain tissue-specific genes in mammals [16] (Figure 1). The genome-wide DNA methylation pattern in all cells of the body is basically bimodal. Centromeric and pericentromeric regions, as well as other repetitive elements are heavily methylated. Many genic regions also show high degrees of methylation. In contrast, CpG islands are largely unmethylated (<10%). Once set up, the basic bimodal methylation pattern is maintained through every cell division and serves as a global repression mechanism. Adjustments are made through targeted *de novo* methylation and demethylation [17].

DNA methylation status is dynamically regulated by DNA methylation and demethylation reactions. There are three active DNA methyltransferases in mammals: DNMT1, DNMT3A, and DNMT3B. DNMT1 maintains DNA methylation at hemi-methylated DNA after DNA replication during cell divisions, whereas DNMT3A and DNMT3B are responsible for establishing *de novo* DNA methylation [18]. A third member of the DNMT3 family, DNMT3-like (DNMT3L), which has no catalytic activity, functions as a regulator of DNMT3A and DNMT3B [19,20]. Mammalian DNMT2 is a tRNA methyltransferase rather than a DNA methyltransferase and has been renamed tRNA aspartic acid methyltransferase 1 [21]. Much of the repressive potential of methylated DNA is elicited by methyl-DNA binding proteins. These proteins bind methylated DNA via a conserved methyl-CpG binding domain (MBD1, MBD2, MBD3, MeCP2 and MBD4) or zinc finger domain (Kaiso, ZBTB4 and ZBTB38) [22], and can recruit remodeling factors such as histone deacetylases and histone H3 lysine 9 methylases which further contribute to repressive chromatin state [16].

The mechanisms underlying active DNA demethylation are less understood. Methyl-CpG-binding domain protein 2 (MBD2) was the first reported protein to carry out enzymatic removal of the methyl group of 5meC. Another model of DNA demethylation proposes modification of 5mC through deamination or hydroxymethylation, reactions catalyzed by Aid, APOBEC or Tet enzymes, respectively. Modified bases are subsequently removed by glycosylases, which generate apyrimidinic acid that is subsequently removed by base excision repair pathway and replaced with cytosine [17].

## 2. Deregulation of the Epigenetic Landscape in Cancerogenesis

### 2.1. Activation or Inactivation of Epigenetic Enzymes in Cancer

Deregulation of the epigenetic landscape can also occur due to activation or inactivation of the enzymes that maintain and modify the epigenome. Epigenetics enzymes are frequent targets for mutation [23]. DNA methyltransferases have been found to be genetically altered, *i.e.*, DNMT3A in acute monocytic leukemia [24,25,26]. It has been suggested that the R882 DNMT3A mutations may alter functions of DNMT3A such as its ability to bind other proteins involved in transcriptional regulation and localization to chromatin regions containing methylated DNA [26]. Ten-Eleven-Translocation 2 (TET2) is an enzyme which catalyzes the conversion of 5-methylcytosine into 5-hydroxymethylcytosine (5-hmC) and thereby influences the epigenetic state of DNA. Loss-of-function TET2 mutations were also identified in 20%–30% myeloid neoplasms [27,28].

Cytogenetic studies, as well as next generation sequencing of various cancer genomes, have demonstrated recurrent translocations and/or coding mutations in a large number of lysine methyltransferases, including MMSET, EZH2, and MLL family members. Follicular lymphomas contain recurrent mutations of the histone methyltransferase MLL2 in close to 90% of cases [29]. The oncogenic effects exerted by the MLL fusions have been extensively studied [30]. Mutations affecting the Polycomb repressive complex (PRC) components, such as EZH2, can also affect histone modifications and have recently been reported. EZH2 is the enzymatic component of the PRC2 complex and is a H3K27 methyltransferase. Overexpression of EZH2 has been reported in prostate [31] and breast [32] cancers, and in several types of leukemia [33,34]. Gene silencing caused by overexpression of EZH2 has been linked to the progression of breast, bladder and prostate cancers [31,32,35]. Recurrent coding mutations have also been noted in histone demethylases KDM5A (JARID1A), KDM5C (JARID1C), and KDM6A (UTX). Mutations in UTX, in particular, are prevalent in a large number of solid and hematological cancers [36].

Although somatic mutations in HDACs do not appear to be prominent in cancer, the expression levels or activities of various HDACs are altered in numerous malignancies. Chimeric fusion proteins occurring in leukemia, such as PML-RARa, PLZF-RARa and AML1-ETO, have been shown to recruit HDACs to mediate aberrant gene silencing that contributes to leukemogenesis [37]. HDACs can also interact with oncogenic proteins such as BCL6, whose repressive activity is controlled by dynamic acetylation [38].

### 2.2. Metabolic Disturbances as a Source of Epigenetic Deregulation

Several recent findings emphasize the interplay between the Krebs cycle, epigenomic changes and cancer [39]. In contrast to normal cells which rely on mitochondrial oxidative phosphorylation to produce energy from glucose, cancer cells prefer to metabolize glucose by glycolysis, resulting in increased glucose consumption and lactate production [40]. Otto Warburg observation, known as the Warburg effect or aerobic glycolysis, has become the basis of ^18^FDG-PET imaging in current clinical practice [41]. Interestingly, mutations in the genes coding for metabolic enzymes such as succinate dehydrogenase (SDH), fumarate hydratase (FH), isocitrate dehydrogenases 1/2 (IDH1/2) and phosphoglycerate dehydrogenase (PHGDH) have been reported in various tumors: paraganglioma [42], renal cancer [43] and glioblastoma multiforme [44,45,46]. Mutations in *IDH1* and *IDH2* are also noted as recurrent mutations in a range of myeloid malignancies, most notably AML [47]. Mutations in *RET*, *NF1* (neurofibromatosis 1), *VHL* (the von Hippel-Lindau) and *SDH* genes are frequent in paragangliomas which are neuroendocrine tumors. Methylome analysis of a large paraganglioma cohort identified three stable clusters, associated with distinct clinical features and mutational status. SDH-related tumors displayed a hypermethylator phenotype, associated with down-regulation of key genes involved in neuroendocrine differentiation. Succinate accumulation in SDH-deficient mouse chromaffin cells led to DNA hypermethylation by inhibition of 2-OG-dependent histone and DNA demethylases and was associated with a migratory phenotype. Epigenetic silencing was particularly severe in SDHB-mutated tumors, potentially explaining their malignancy [48]. Together these findings indicate that the metabolic switches are not just byproducts of cancer development, but major contributors to it.

## 3. Epigenetic Modifications in Glioblastomas

Gliomas, the most common tumors of the central nervous system, represent over 70% of all brain malignancies. The grade of a particular glioma is defined by World Health Organization (WHO) criteria based on histological assessment for atypical cells, mitoses, endothelial proliferation, and necrosis. Pilocytic astrocytoma (PA, WHO grade I) is a slow growing tumor that usually does not spread to surrounding brain tissue [49]; while glioblastoma multiforme (GBM, WHO grade IV) is the most aggressive, highly diffusive and vascularized glioma. Despite recent advances in surgery, radiotherapy and chemotherapy, survival of glioma patients remain poor and the mean survival is only 12 to 15 months. Glioblastomas are considered to be one of the most difficult human malignancies to treat due to frequent dysfunctions of tumor suppressors and oncogenes [50,51].

Recent studies characterizing different GBM subgroups with regard to gene expression [52,53,54], DNA methylation [55] or miRNA expression [56] profiles indicate that besides genetic alterations, epigenetic modifications could be involved in the development and progression of cancer.

The contribution of epigenetic changes to glioblastoma pathology has been broadly studied in terms of aberrant promoter methylation-induced gene silencing. Candidate gene approaches have demonstrated the hypermethylation of notable genes in GBM, including tumor suppressors, apoptosis machinery, suppressor of cytokine signaling family, members of Wnt-signaling pathway and hypomethylation of normally silenced genes such as CD133, MMP9 or IL8 (reviewed in [57]) (Table 1). To date, the strongest evidence that epigenetic alterations are associated with patient survival is provided by the methylation-associated silencing of the *MGMT* gene in gliomas. The *MGMT* gene encodes DNA repair protein O^6^-methylguanine-DNA methyltransferase (MGMT). *MGMT* hypermethylation is the best independent predictor of glioma patients’ response to alkylating agents, such as temozolomide [58].

**Table 1:** Genes abnormally methylated in glioblastoma.

DNA methylation	Function	Gene	References
**Hypermethylation**	DNA repair	MGMT	[57]
	Tumor suppressors	RB, HIC1, CDKN2A, p14, p16INK4, PTEN, RRP22, TP53, TES, BEX1, BEX2, BLU	[57,59,60,61,62,63]
	Cell proliferation	EMP3	64
	Apoptosis	RASFF1A, CASP8, TNFRSF10A, TMS1	[57,62,63]
	Suppressors of cytokine signaling	SOCS1, SOCS2, SOCS3	[57]
	Wnt signaling	SFRP1, SFRP2, NKD2	[57]
	Transcription factors	GATA6, HOXA, RFX1, RUNX3	[61,63,65]
**Hypomethylation**	Epigenetics	DNMT3B	[66]
	Invasiveness	MMP9	[57]
	Stemness	CD133	[67]
		IL8, POTEH, IGF2	[57,68,69]

Recent findings related to recurrent mutations in the genes encoding the metabolic enzymes IDH1 and IDH2 have broad implications for the Jumonji class of demethylases, which use a-ketoglutarate (a-KG). IDH1/2 are nicotinamide adenine dinucleotide phosphate (NADP)-dependent enzymes which normally catalyze the oxidative decarboxylation of isocitrate to a-KG, associated with the production of NADPH. These mutations manifest in a neomorphic enzymatic activity which results in the NADPH-dependent reduction of a-KG to 2-hydroxyglutarate (2-HG). 2-HG has been shown to adopt a near-identical orientation within the catalytic core of the JmjC domain and DNA hydroxylases [70]. Accumulation of 2-HG within the malignant cells may lead to complete inhibition of the Jumonji class of histone demethylases as well as DNA demethylation process. Alternative mechanisms include: increased angiogenesis due to accumulation of hypoxia inducible factor (HIF)-1α; a glioma CpG island methylator phenotype induced by inhibition of TET2, and increased vulnerability to oxidative stress due to depletion of antioxidants [71,72]. The intensive influence of the CpG island methylator phenotype on gene expression suggests that this may result from a defect in a transcription factor involved in the protection of a defined subset of CpG island promoters from DNA methylation. Loss of function of this factor would result in widespread DNA methylation changes [55].

One of such factors could be the nuclear receptor SET domain containing protein-1 (*NSD1*) gene, which encodes a histone methyltransferase involved in chromatin regulation. Loss-of-function mutations and deletions of *NSD1* gene have been found in Sotos syndrome, which is an autosomal dominant condition characterized by overgrowth (tall stature and macrocephaly) and an increased risk of tumorigenesis. NSD1 function was found abrogated by transcriptional silencing associated with CpG island-promoter hypermethylation in human neuroblastomas and gliomas [73]. The epigenetic inactivation of NSD1 in transformed cells resulted in diminished methylation of the histone lysine residues H4-K20 and H3-K36 [73].

The *IDH1* or *IDH2* genes are mutated in 50%–80% of astrocytomas, oligodendrogliomas or oligoastrocytomas of grades II and III, and secondary glioblastomas. *IDH1* and *IDH2* mutations are heterozygous, affect only a single codon: the 132 amino acid in the *IDH1* and the analogous amino acid (172) of the IDH2 gene, and rarely occur together [74]. Higher IDH1 mutation rates are seen in grade II and III astrocytomas and oligodendrogliomas [75,76]. However, they are seldom mutated in primary glioblastomas and never in other types of glioma. Similar results were reported by Hartmann *et al.* [77], who detected 716 *IDH1* mutations and 31 *IDH2* mutations (Table 2). The pilocytic astrocytomas and gangliogliomas did not have either mutation [78]. Gliomas with *IDH1/2* mutations always harbor either *TP53* mutations or total 1p/19q loss [72]. Patients with the CpG island methylator phenotype tumors are younger at the time of diagnosis and show significantly improved outcome [55].

Glioblastomas with the CpG island methylator phenotype constitute a subset of tumors with extensive epigenomic aberrations and distinct biology [55]. Assessment of the epigenome of a large set of intermediate-grade gliomas demonstrates a CpG island methylator phenotype which is highly dependent on the presence of *IDH* mutation (IDH1-R132). Introduction of mutant *IDH1* into primary human astrocytes alters specific histone marks, induces extensive DNA hypermethylation, and reshapes the methylome in a fashion that resembles the CpG island methylator phenotype in low-grade gliomas. The epigenomic alterations produced by a mutant IDH1 activate gene expression programs resembling proneural glioblastomas but not other glioblastomas [79].

**Table 2:** Frequency of *IDH1/2* mutation in gliomas.

Tumor classification		*IDH1* mutation (%)		*IDH2* mutation (%)	
		Yan *et al*. [74]	Hartman *et al*. [77]	Yan *et al*. [74]	Hartman *et al.* [77]
Astrocytic tumors	Pilocytic astrocytoma (grade I)	0.0	-	0.0	-
	Subependymal giant-cell astrocytoma (grade II)	0.0	-	-	-
	Diffuse astrocytoma (grade II)	83.3	72.7	6.6	0.9
	Pleomorphic xanthoastrocytoma (grade II)	14.0	-	-	-
	Anaplastic astrocytoma (grade III)	69.2	64.0	3.8	0.9
	Secondary glioblastoma (grade IV)	85.0	-	0.0	-
	Primary adult glioblastoma (grade IV)	5.0	-	0.0	-
	Primary pediatric glioblastoma (grade IV)	0.0	-	0.0	-
Oligodendroglial tumors	Oligodendroglioma (grade II)	80.3	82.0	3.9	4.7
	Anaplastic oligodendroglioma (grade III)	86.1	69.5	8.3	5.2
Oligoastrocytic tumors	Oligoastrocytoma (grade II)	100.0	81.6	-	1.3
	Anaplastic oligoastrocytoma (grade III)	100.0	66.1	-	6.2

As *IDH1/2* mutations are frequent (>80%) in secondary glioblastomas that have progressed from low-grade or anaplastic astrocytomas, it suggests that these tumors share a common progenitor cell population. Primary glioblastomas with *IDH1/2* mutations are very rare (<5%); show age distribution and genetic profiles similar to secondary glioblastomas, therefore could be misclassified. Although histologically similar, GBMs arising with and without IDH1(R132MUT) exhibit several features, including frontal location and lesser extent of contrast enhancement and necrosis, which distinguish IDH1(R132MUT) GBMs from other GBMs. It relates IDH1(R132MUT) GBMs to lower-grade IDH1(R132MUT) gliomas [80]. Methylation profiling revealed that GBM samples with low grade glioma-like hypermethylated profiles had a high rate of IDH1 mutations and a better outcome [81]. Therefore, IDH1(R132MUT) GBMs appear to represent distinct disease entities which arise from distinct cell types of origin as the result of non-overlapping molecular events [80]. The concept of sequential molecular evolution of the IDH1 mutant glioblastoma has been conceived. The observed patterns of DNA changes, DNA methylation, and copy number alterations suggest an ordered molecular evolution of IDH1(R132MUT) GBM in which the appearance of mutant IDH1 protein in spatially and temporally restricted neural precursors is an initial event, followed by production of TP53 mutant protein, and finally by copy number alterations of *PTEN* and *EGFR* [80].

Mechanisms of epigenetic deregulation dependent on histone modifications are less well characterized. However, immunohistochemical analysis of the global pattern of H3K4me2, H4K20me3, H3K9Ac and H3K18Ac in resected 230 glioma samples showed that histone modifications may have prognostic relevance in gliomas, as some correlations with progression free- and overall survival of glioma patients were reported. Histone modification pattern divided primary and secondary glioblastoma groups, with the former having a better prognosis. Acetylation of H3K18 significantly influenced survival of primary glioblastoma patients, with a greater survival for patients whose tumor expressed lower levels (<74% of tumor cells) of H3K18Ac. H4K20me3 significantly influenced survival of secondary glioblastoma patients, with a greater survival of patients whose tumor expressed higher H4K20me3 levels (≥75% of tumor cells) [82]. Trimethylation of histone 3 lysine 9 (H3K9me3) is a marker of repressed transcription. In a recent immunohistochemical study, H3K9me3 positivity was found in all grades of astrocytic tumors and showed significant relationship with the IDH mutational status in grade II astrocytomas but not in grade III astrocytomas or glioblastomas [83].

Until now there is some evidence for deregulation of the histone modifying enzymes in GBM. In particular, the expression of genes coding for histone remodeling complex proteins BMI1, EZH2, which regulate H3K27 methylation, as well as histone deacetylases (HDACs), was found to be altered in a subset of human GBM [84,85,86,87,88]. The levels of class II and IV HDACs mRNAs were downregulated in glioblastomas compared to low-grade astrocytomas and normal brain tissue (seven out of eight genes). The protein levels of class II HDAC9 were also lower in high-grade astrocytomas than in low-grade astrocytomas and normal brain. The levels of acetylated histone H3 (but not histone H4) were higher in glioblastomas than normal brain tissue [88].

The histone methyltransferase Enhancer of Zeste 2 (EZH2) is a key member of the Polycomb repressive complex 2 (PRC2) which functions as an epigenetic gene silencer. Quantitative PCR and immunohistochemistry demonstrated that EZH2 is more expressed in GBM than in low-grade gliomas [86]. Some evidence implicated polycomb group proteins in regulating pluripotency and survival of cancer stem cells. PRC2 mediates lysine 27 trimethylation on histone H3 and in glioma stem cells it affects pluripotency or development-associated genes (e.g., Nanog, Wnt1, and BMP5) [89]. EZH2 expression was detected in tumor cells by immunohistochemistry in paraffin-embedded samples of GBM (five of five samples); no detectable expression was observed in adjacent brain parenchyma. Abrogation of EZH2 function by the treatment with 3-deazaneplanocin A (DZNep) induced transcriptome changes consistent with the role of EZH2 in epigenetic regulation of gene expression. Pharmacologic and shRNA-mediated depletion of EZH2 in glioblastoma cancer stem cells reduced their ability to form new spheres *in vitro* and new tumors *in vivo* [90]. A recent study shows that the number of tumor cells with nuclear localization of EZH2 is larger around tumor vessels and the invasive front in human glioma specimens, suggesting that nuclear EZH2 could contribute to glioblastoma-induced angiogenesis and invasion [91].

Two recent studies have demonstrated recurrent somatic mutations in genes encoding the replication-independent histone H3 variant H3.3 (H3F3A) and the canonical histone H3.1 (HIST1H3B) in 30% of pediatric glioblastomas and only 3.4% of adult GBM [92,93]. These heterozygous mutations are clustered and result in amino acid substitutions at two critical residues in the tail of histone H3 (K27M, G34R/G34V). The H3 K27M mutation alters the ability of this critical residue to be both methylated and acetylated. The G34 mutations, due to their proximity to H3K36, likely influence transcription. Tumors carrying the H3 K27M and G34R/G34V mutations had globally diminished trimethylation at histone H3 K27, distinct gene expression profiles. Tumors with the H3 G34V mutation demonstrated a global increase in H3K36me3 [92]. H3.3K27M mutations alone are not transforming and H3.3K27M is unable to promote glioma even in a p53 null background [94], suggesting that other genetic events are needed. The mutant histone H3 may act as a selective inhibitor of the PRC2 chromatin-modifying complex by binding and presumably sequestering it contributes to the etiology of pediatric glioblastomas [95].

Mutations in *ATRX* (α-thalassaemia/mental retardation syndrome X-linked) and *DAXX* (death-domain associated protein) genes that encode two subunits of a chromatin remodeling complex required for H3.3 incorporation at pericentric heterochromatin and telomeres, were identified in 31% of pediatric GBMs. Tumors with mutations in the *ATRX/DAXX* genes harbored a G34R or G34V H3.3 mutation in a majority of cases. Tumors with mutations in *H3F3A/ATRX/DAXX* genes were associated with the increased length of telomeres and genomic instability [92]. Another study using whole exome sequencing in four low grade astrocytomas, followed by focused resequencing in an additional 28 samples found a high incidence of mutations in the *ATRX* gene [96]. ATRX mutations were entirely restricted to IDH-mutant tumors, closely correlated with TP53 mutation and astrocytic differentiation, and mutually exclusive with 1p/19q co-deletion, the molecular hallmark of oligodendroglioma.

The protein encoded by *FUBP1* (Far Upstream Element [FUSE] Binding Protein 1) binds to single stranded DNA, in particular the FUSE of *MYC*, and in a complex with PUF60 negatively regulates *MYC* expression. FUBP1 mutations may lead to activation of a *MYC* oncogene [97]. The HMG-box protein Capicua (CIC) is a key sensor of receptor tyrosine kinase (RTK) signaling in Drosophila and mammals. Several studies have shown that CIC functions as a repressor of RTK-responsive genes, keeping them silent in the absence of signaling [98]. The mutational analysis of 363 brain tumors reported the distribution of *ATRX*, *CIC* (homolog of the *Drosophila capicua* gene), and *FUBP1* mutations in gliomas [99]. ATRX was frequently mutated in grade II-III adult astrocytomas (71%), oligoastrocytomas (68%), and secondary glioblastomas (57%). ATRX mutations were found to be associated with IDH1 mutations and with an alternative lengthening of telomeres. CIC and FUBP1 mutations occurred frequently in oligodendrogliomas (46% and 24%, respectively) but rarely in astrocytomas or oligoastrocytomas (10%). This analysis defined two highly recurrent genetic signatures in gliomas: IDH1/ATRX (I-A) and IDH1/CIC/FUBP1 (I-CF). Patients with I-CF gliomas had a significantly longer median overall survival (96 months) than patients with I-A gliomas (51 months) and patients with gliomas that did not harbor either signature (13 months) [99].

RRP22 (Ras-related protein on chromosome 22) has been suggested as a candidate tumor suppressor in human cancers. Its expression was decreased in high-grade gliomas (WHO grades III and IV) compared with low-grade gliomas (WHO grade II) and was associated with shorter overall survival in 180 glioblastoma patients included in the NIH REMBRANDT database. Decreased RRP22 expression was in part explained by 5'-CpG island hypermethylation and acetylated histone H3 and H4. In primary human glioblastomas, the increased levels of H3K9me3-bound and the decreased levels of pan-Ac-H3-bound RRP22 were observed as compared to non-neoplastic brain tissue. It shows that 5'-CpG island hypermethylation and histone modifications may contribute to the frequent and prognostically unfavorable transcriptional down-regulation of RRP22 in malignant gliomas [60].

## 4. Epigenetic Inhibitors as Potential Anti-Glioblastoma Therapeutics

As epigenetic modifications by its nature are reversible, therefore changes in the epigenome associated with cancer are potentially reversible. It opens up the possibility of using “epigenetic drugs” which may have a powerful impact on various cancers, including glioblastomas. A number of small-molecule inhibitors against chromatin regulators have been developed and some of them are already used in clinical cancer treatment or are currently in preclinical and clinical trials (Table 3). Indeed, HDAC inhibitors have been found to be particularly effective in inhibiting tumor growth, promoting apoptosis and inducing differentiation [100,101], at least in part via the reactivation of certain tumor suppressor genes. Two demethylating agents, *i.e.*, inhibitors of DNA methyltransferase (5-azacytidin and decitabin) were approved by the Food and Drug Administration (FDA) in the treatment of myelodysplastic syndrome and a few inhibitors of histone acetylases (vorinostat, romidepsin and panobinostat) are approved in the treatment of hematological malignancies, particularly in refractory or relapsed cutaneous T-cell lymphoma. Other compounds are presently in phase II and III clinical trials [102,103]. Hypomethylating agents are also one of the few epigenetic therapies that have gained FDA approval for routine clinical use. Small-molecule inhibitors of histone demethylases are at various stages of development and emerging preclinical data show the therapeutic potential of compounds.

**Table 3:** Small-molecule inhibitors in preclinical and clinical trials for glioblastoma treatment.

Experiment	Enzyme	Inhibitor	References
***in vitro***	HDACs	Vorinostat (SAHA), PCI-24781, TSA, VPA, Scriptaid, MS-275, AR42	[104,105,106,107,108,109,110,111,112]
	HAT	curcumin	[113]
	LSD1	tranylcypromine	[109]
	DNMT	5-azacytidine, 5-aza-2'-deoxycytidine, zebularine, psammaplin A	[114]
	EZH2	3-deazaneplanocin	[67]
***in vivo***	HDACs	Vorinostat (SAHA), TSA, VPA, MS-275	[104,107,111,115,116]
	DNMT	5-azacytidine	[115]

To date, there are a few studies testing the impact of the histone modifying enzymes inhibitors on glioblastomas, some of them show promising results. For example the histone deacetylation inhibitor, suberoylanilide hydroxamic acid crossed the blood-brain barrier, inhibited the proliferation of intracranial gliomas and increased mice survival [117]. The AGI-5198 inhibitor identified through a high-throughput screen as a selective R132H-IDH1 inhibitor, induced demethylation of histone H3K9me3 and expression of genes associated with gliogenic differentiation. Blockade of a mutant IDH1 impaired the growth of IDH1-mutant, but not IDH1-wild-type, glioma cells [118]. Interestingly, inhibition of a mutant IDH1 did not induce detectable changes in genome-wide DNA methylation that suggests additional growth supporting mechanisms beyond well-characterized epigenetic effects of a mutant IDH1. The study provided a proof-of-concept that mutant IDHs are therapeutically targetable, and that their effects are reversible. Understanding the importance of epigenetic alterations in pathobiology of glioblastomas, and proof-of-concept studies showing that epigenetic modifiers are therapeutically drugable will drive intensive preclinical and clinical studies in search for novel therapeutics in glioblastoma therapy.

## 5. Conclusions

Occurrence of somatic mutations in genes encoding the replication-independent histone H3 variant H3.3 and the canonical histone H3.1, the presence of mutant IDH1 protein in spatially and temporally restricted neural precursors, deregulated expression/activity of epigenetic enzymes may lead to aberrant histone modification and DNA methylation profiles which are intimately linked to glioma pathology. Growing evidence demonstrates a variety of mechanisms, all of which lead to global deregulation in the epigenetic landscape in glioblastomas and support an idea of glioblastoma being an epigenetic malignancy. The high frequency (>80%) of *IDH1/2* mutations in secondary glioblastomas which have progressed from low-grade gliomas, suggests that these tumors share a common progenitor cell population which through sequential molecular evolution gives rise to the IDH1 mutant glioblastoma. The observed patterns of DNA changes, DNA methylation and copy number alterations suggest molecular evolution in which the appearance of mutant IDH1 protein in spatially and temporally restricted neural precursors is an initial event, followed by production of TP53 mutant protein, and finally by copy number alterations of *PTEN* and *EGFR* [80]. It suggests a causative role of epigenetic deregulation in pathobiology of glioblastomas.

Unlike DNA mutations, changes in the epigenome associated with cancer are potentially reversible, which opens up the possibility that “epigenetic drugs” may have a powerful impact within the treatment regiments of various cancers. Though, there are a few studies testing the impact of epigenetic inhibitors on glioblastomas, some of them show promising results and undoubtedly will lead to further preclinical and clinical studies of those compounds in glioblastoma therapy.
